# *Waxy* allele diversification in foxtail millet (*Setaria italica*) landraces of Taiwan

**DOI:** 10.1371/journal.pone.0210025

**Published:** 2018-12-31

**Authors:** Shu-meng Kuo, Yu-ru Chen, Song-yu Yin, Qing-xiong Ba, Yuan-ching Tsai, Warren H. J. Kuo, Yann-rong Lin

**Affiliations:** 1 Department of Agronomy, National Taiwan University, Taipei, Taiwan; 2 Crop Science Division, Taiwan Agricultural Research Institute, Taichung, Taiwan; 3 Department of Agronomy, National Chiayi University, Chiayi, Taiwan; National Institute of Plant Genome Research, INDIA

## Abstract

Foxtail millet (*Setaria italica* (L.) P. Beauv.), the second most cultivated millet species, is well adapted to diverse environments and remains an important cereal food and forage crop in arid and semiarid regions worldwide. A symbolic crop for indigenous Austronesian peoples, foxtail millet has been cultivated in Taiwan for more than 5,000 years, and landraces reflect diversifying selection for various food applications. A total of 124 accessions collected within Taiwan were assessed for *Wx* genotypes. Four identified *Wx* alleles, I, III, IV, and IX were caused by insertion of various transposable elements (TEs) and resulted in endosperm with non-waxy, low amylose content (AC), and waxy, respectively. A total of 16.9%, 4.0%, 49.2%, and 29.8% of accessions were classified as type I, III, IV, and IX, respectively; approximately half of the accessions belonged to the waxy type, indicating that glutinous grains were favored for making traditional food and wine. The TE insertion affected splicing efficiency rather than accuracy, leading to significantly reduced expression of *wx* in types III, IV, and IX, although their transcripts were the same as wild-type, type I. Consequently, the granule-bound starch synthase I (GBSSI) contents of the three mutated genotypes were relatively low, leading to waxy or low AC endosperm, and the *Wx* genotypes could explain 78% of variance in AC. The geographic distribution of *Wx* genotypes are associated with culinary preferences and migration routes of Taiwanese indigenous peoples—in particular, the genotype of landraces collected from Orchid Island was distinct from those from Taiwan Island. This information on the major gene regulating starch biosynthesis in foxtail millet endosperm can be applied to breeding programs for grain quality, and contributes to knowledge of Austronesian cultures.

## Introduction

Population growth and climate change both threaten to agricultural productivity. Noting that the world population is highly dependent on a cereal-based diet with chronic micronutrient deficiency, enhancement of crop and nutritional diversity by developing resource use-efficient crops and accelerating biofortification is important to food security [1]. Foxtail millet (*Setaria italica* (L.) P. Beauv), with whole grain composed of 3.3% ash, 6.7% crude fiber, 4.0% crude fat, 11.2% crude protein, 63.2% starch, and 12% moisture, is highly nutritious in comparison with major cereals such as rice and wheat [2]. Its lysine richness suggests the possible use of foxtail millet as a supplementary protein source to most cereals, and the majority of its crude fat is unsaturated, such as linoleic acid [1, 2]. Furthermore, with a short growing season, good productivity on barren soils and adaptation to extreme environments such as drought tolerance, foxtail millet is a preferred choice for improving food and nutritional security in the semi-arid tropics. Foxtail millet ranks second in total world production among millets, cultivated as staple food in semi-arid tropics of Asia such as China and India, and as fodder and hay in Europe, North America, Australia, and North Africa [3, 4].

Foxtail millet is an annual grass and self-pollinating species with a relatively short life cycle, small plant stature, abundant seed production, diploidy (2n = 2x = 18) with a small genome (~515 Mb), and high genetic diversity [5–8]. Because foxtail millet and its wild progenitor, green foxtail, are easy to grow under low-input conditions and transformable as well, they have recently become a potential C_4_ model for polyploid relatives such as switchgrass (*Panicum virgatum* L.) and to address biological questions related to abiotic stress tolerance and the evolution of C_4_ photosynthesis [5, 9–12].

The domestication of foxtail millet dates to ~6000 BC, with four major cultivated regions, China, Central Asia, Europe and the Near East, suggested as possible domestication centers [13]. Recent studies tend to support the hypothesis that foxtail millet domestication was monophyletic [14]. In Taiwan, carbonized and charred seeds of foxtail millet unearthed in the Nan-kuan-li East site indicated that its cultivation dates to ~5,000 years ago [15]. Taiwanese indigenous peoples, Austronesian language speakers presently consisting of 16 ethnic groups, cultivate foxtail millet not only for staple food but for various delicacies including millet cakes, wine, and seasonings, that are served in ritual feasts and special occasions such as harvest festivals and weddings. Today, instead of declining like other millets, foxtail millet remains prestigious as a symbolic crop and food with more than 160 landraces recorded since the 1960’s [16]. By cluster analysis with molecular markers, Taiwanese foxtail millet landraces were divided into three groups which were consistent with northern, central, and southern areas in Taiwan; additionally, the population structure and genetic diversity coincided with indigenous migration [16].

As the major component of foxtail millet grain and other cereals, starch determines ultimate utilization of foxtail millet. In foxtail millet endosperm, amylose and highly branched amylopectin account for 20–30% and 70–80% of total starch, respectively. Amylose content (AC) is highly correlated with enzyme activity of granule-bound starch synthase I (GBSSI), encoded by the *Waxy* (*Wx*) gene. Gene structures of non-waxy (wild-type) alleles in cereal crops, e.g. foxtail millet, sorghum, maize, and rice, share high similarity. Nevertheless, their *waxy* alleles are quite diverse because of mutations such as SNPs, small indels, or transposable element insertions, reducing expression of *Wx* and/or causing nonsynonymous mutations with consequently decreased and/or altered GBSSI activities [17]. In rice, *Waxy* alleles are mainly caused by single nucleotide polymorphisms (SNPs). For instance, *Wx*^*b*^, the common allele in *japonica* rice, caused by alternative splicing of intron 1 that further affects the levels of mature *Wx* messenger RNA (mRNA), Wx protein (OsGBSS1), thus leading to low (12–19%) AC in comparison with the wild-type allele, *Wx*^*a*^ (22–29%) [18]. Seven representative haplotypes were identified from rice pan-genomes constituted from 66 rice accessions, revealed that 23-bp duplication at the second exon caused glutinous grains in addition to G/T at the intron 1–exon 1 junction site [19]. Alleles resulted from SNPs uncovered from landraces, e.g. *Wx*^*op*^, *Wx*^*mq*^, and *Wx*^*hp*^, or mutated accessions affected AC, grain appearance, and eating and cooking quality as consequence [20–23]. In barley, the level of *GBBSI* mRNA was greatly reduced by the deletion of the promoter and/or 5’UTR [24, 25]. In maize, TE insertion altered *Wx* expression and reduced AC such as two alleles identified in Chinese maize landraces, *wx-Cin4* and *wx-124* [26, 27], and low AC were also brought by deletion mutations [28, 29]. In wheat, inactive GBSSI was the outcomes of by SNPs, TE insertions, indels occurring at different *Wx* positions [30–32]. In sorghum, TE insertion and SNPs lead to four waxy alleles: *wx*^*a*^ (~1.63% AC), *wx*^*b*^ (~1.45%), *wx*^*c*^, and *wx*^*d*^ (0.71% AC) [33, 34]. In Job’s tears (*Coix lacryma-jobi* L), a 275-bp deletion in exons 10 to 11 of *Wx*, leading to the lack of GBSSI protein, was found specific to Japanese and Korean waxy cultivars [35]. Broomcorn, proso, or common millet (*Panicum miliaceum* L.), an allotetraploidy cereal, has two homeologous *Wx* loci that *GBSSI*-S locus is the major locus while *GBSSI-L* has reduced enzyme activity. The genotype combinations of *GBSSI-S* and *GBSSI-L* alleles resulted from deletion, frameshift, and SNPs contributed to AC variation in cultivars and landraces corresponding to waxy endosperm. [36, 37].

In foxtail millet, *Wx*, located on chromosome 4, is 4,211 bp long and contains fourteen exons with exons 2 to 14 encoding 605 amino acids. Thirteen alleles arisen from insertions of various TEs have been identified by PCR amplification [38]. Of these, types I and II are non-waxy; types III, II/VI (intragenic recombination), VI and IX are low AC; and types V, VII, VIII, X, and IV, including two derived types, IVa and IVb, are waxy. The hypothesis that waxy endosperm of foxtail millet might have evolved from the non-waxy wild-type after domestication, is supported by two facts–(1) the wild progenitor of foxtail millet, green foxtail, possessed a non-waxy endosperm; and (2) *Wx* DNA sequences of non-waxy accessions were more diverse than those of low amylose and waxy types [17, 38]. The AC of waxy endosperm is quite low or near 0% because of reduced expression or loss of function of *Wx* (*wx*), whereas the dominant *Wx* allele is associated with > 10% AC. According to AC variations, foxtail millet was classified into three types, namely non-waxy (17.0 to 31.9%), low-AC (7.8 to 16.0%) and waxy (0 to 3.5%) [39]. Waxy type landraces of foxtail millet are mainly cultivated in East Asia and Southeast Asia [39–41].

In the present study, a diversity panel of 124 foxtail millet accessions, including 8 varieties and 116 landraces collected from different tribes and regions in Taiwan, was subjected to AC and *Wx* genotype analyses. The impact of large TE insertions on AC was evaluated at both transcriptional and translational levels. This study provides new evidence regarding how *Wx* alleles affect expression profiles during grain filling and AC with consequences for foxtail millet. An intriguing inter-related question regards whether the geographic distribution of *Wx* genotypes could reflect the selection preferences of different ethnic groups.

## Materials and methods

### Plant materials

A diversity panel of 124 accessions of Taiwan foxtail millet used in this study (S1 Table). The 92 accessions designated with PI numbers that were collected by Wayne Fogg and deposited in the United States Department of Agriculture (USDA) in 1970s, were introduced back to Taiwan and deposited in the National Plant Genetic Resources Center (NPGRC), Taiwan Agriculture Research Institute (TARI) by the two co-authors, Qing-xiong Ba and Warren H. J. Kuo. Two accessions, TTS-1 and TTS-5, were requested from Taitung District Agricultural Research and Extension Station (TTDARES), Council of Agriculture, Taiwan. The other 30 accessions were collected without any inappropriate ethic concerns because foxtail millet is neither endangered nor protected species. In addition, the collection sites of these 30 accessions are not in indigenous territories for which no specific permissions are required. All accessions were cultivated for two crop seasons, 2015 and 2016, in a randomized complete block design (RCBD) with three replications in the TARI experimental farm.

### Measurement of apparent amylose content (AAC)

AAC was determined by an iodine-staining (I_2_-KI) assay [42]. The amylose standard solution was prepared by placing 40 mg of potato amylose (Sigma, USA) in a 1.5 mL eppendorf tube then adding 50 μL of 95% ethanol and 450 μL of 1 M NaOH. Amylose standard solution was then diluted to 0%, 10%, 20%, 30%, and 40% to obtain a standard curve. Each sample was diluted with 1 mL distilled water to a final volume of 1.5 mL and followed the same preparation method as the standard solution. A total of 5 μL sample was then mixed with 295 μL staining solution (5 mL H_2_O, 0.5 mL 1 M acetate, and 0.5 mL iodine solution of 2% potassium iodide and 0.2% iodine). The AAC of each sample was calculated by the standard curve based on absorption at O.D. 620 nm.

### Genotyping assay of *Wx*

Total genomic DNA was extracted from 2-week old seedling leaves using a cetyl trimethylammonium bromide (CTAB) method with some modifications [43]. Amplicons larger than 1 kb were amplified by long range PCR using LA Taq DNA polymerase with GC buffer system (TaKaRa Bio Inc., Japan). *Wx* alleles were identified by using three primer pairs adopted from studies of Fukunaga et al. (2002) and Kawase et al. (2005) (ex1 and ex2, ex2int2 and ex4r, M7 and R9 [38, 40]) and one pair designed in this study (ex7 and ex10) (S2 Table). PCR products were analyzed by electrophoresis using 1.0% agarose gels at constant voltage of 120 V for 45 minutes.

### *Wx* gene expression analyzed by quantitative real time PCR (qRT-PCR)

Expression levels of *Wx* during endosperm development were analyzed at 5, 10, 15, and 22 days after flowering (DAF). Because inflorescences of *Setaria* species have multiple orders of branching and flowers on the different branches open at different times, we recorded the day of anthesis as the beginning of flowering and to study endosperm development we sampled the same parts of the inflorescence where anthesis was first observed [44]. Total RNA was extracted using Direct-zol RNA MiniPreps (Zymo Research Corp, USA) and was applied to generate the first strand of cDNA with PrimerScript RT Reagent Kits (TaKaRa Bio Inc., Japan). The first strand cDNA was amplified in a 20 μl qRT-PCR reaction (KAPA SYBR FAST qPCR Kit Master Mix, KAPA Biosystems, USA). For each sample, three biological replicates of qRT-PCR reactions were performed with *Elongation factor 1-alpha* (*EF1a*) as the internal control. The newly designed primers were for both *GBSSI* (F: ctccacaccaccaccaaggg/ R: gcagctggttgtccttgtaa) and *EF1a* (F: cactcttggagtgaagcagatgatc/ R: cgaaggcaatcttgtcagggttgta) in qRT-PCR and RT-PCR. Significant differences in expression levels of *Wx* were determined by using two-way analysis of variance (ANOVA) and the Fisher’s Least Significant Difference (LSD) test at *p* < 0.05 level by R (version 3.4.2).

### The measurement of starch granule-bound protein content

Starch granule-bound proteins were prepared from purified starch according to published methods with some modifications [45]. Developing endosperm samples harvested at 15 DAF were homogenized and suspended in pre-chilled soluble protein extraction buffer (50 mM Tris-HCl, pH8.0, 2 mM EDTA, 10% glycerol, 100 mM NaCl and 1mM phenylmethylsulfonyl fluoride). The homogenate was centrifuged at 13,000 g for 20 min at 4°C, and the pellet containing granule-bound proteins was saved. After resuspending the pellet, proteinase K was added to 50 μg/mL and incubated at 37°C for 30 minutes. Proteinase K was eliminated by washing the granules with extraction buffer once. The starch was washed with 1 mL of ethanol, then with 1 mL of acetone, then dried. Starch granules were boiled for 10 minutes in protein denaturing extraction buffer (50 mM Tris-HCl, pH6.8, 10% glycerol, 5% SDS, 5% β-mercaptoethanol) at a ratio of 15 μL/mg starch. The gelatinized starch was centrifuged at 13,000 g for 15 min. The supernatants containing proteins were precipitated with 3 volumes of acetone. The protein pellet was dried then resuspended in 100 μL of protein extraction buffer. Protein concentration was determined by the Bradford assay using Coomassie Plus Protein Assay Reagent (Bio-Rad, USA). Proteins were separated by SDS-PAGE using 12% (w/v; resolving gel) and 5% (w/v; stacking gel) acrylamide. Electrophoresis was carried out in Tris–glycine SDS electrophoresis buffer at constant voltage of 100 V for 90 minutes. The gel was then electro-blotted onto a polyvinylidene difluoride membrane in transfer buffer (0.01 M Tris, 0.1 M glycine, 20% methanol) at constant voltage of 70 V on a semi-dry transfer cell for 75 minutes (Trans-Blot, Bio-Rad). The blot was blocked with 5% (w/v) dry milk in PBST (80 mM Na_2_HPO_4_, 20 mM NaH_2_PO_4_, 100 mM NaCl, and 0.1% (v/v) Tween-20) and incubated with antiserum (anti-SbWx protein antiserum, 1/3,000), then washed three times in PBST, incubated with secondary antibody (goat anti-rabbit IgG; 1:5,000), conjugated with horseradish peroxidase, and washed again [46]. The signal was detected by the ECL Plus System (GE Healthcare, USA) and quantified by using ImageJ (National Institutes of Health, USA). The amounts of starch granule-bound proteins for different *Wx* genotypes were compared by using the Fisher’s Least Significant Difference (LSD) test at a significance threshold of *p* < 0.05, implemented in R (version 3.4.2).

### Applications of foxtail millet in Taiwanese indigenous peoples

An unstructured interview and participant observation by participating the cultivation were performed to conduct the survey of foxtail millet application in Taiwanese indigenous peoples. One of the coauthors, Qing-Xiong Ba from Rukai people, visited tribes and interviewed elderly aged over 70 to know the applications of foxtail millet by the native language names, plant morphology, harvested panicles and grains. Twenty-four elderlies from 12 tribes belonged to 5 ethnic groups were interviewed (Table 1).

**Table 1:** Applications of foxtail millet in Taiwan’s indigenous foods and cultures.

Ethnic group	Region ^a^	Application								
		Dumpling	Pickling meat	Porridge	Steamed grain	Sticky Cake	Wine	Ancestor worship	Divination	Gift
Atayal	Ren’ai, Nantou (1, 2)		×	×	×	×	×	×		
Bunun	Haiduan, Taitung (2, 4); Shinyi, Nantou (3, 6)			×			×	×		×
Paiwan	Daren, Taitung (1, 2); Dawu, Taitung (1, 2)	×		×	×	×	×	×		×
Rukai	Wutai, Pingtung (2, 4)	×		×	×	×	×	×	×	×
Tao	Orchid Island, Taitung (1, 2)							×		×

^a^ numbers in parentheses indicate the number of communities interviewed (left) and people interviewed (right).

## Results

### Identification of *Wx* genotypes of foxtail millet germplasm from Taiwan

**Fig 1:**
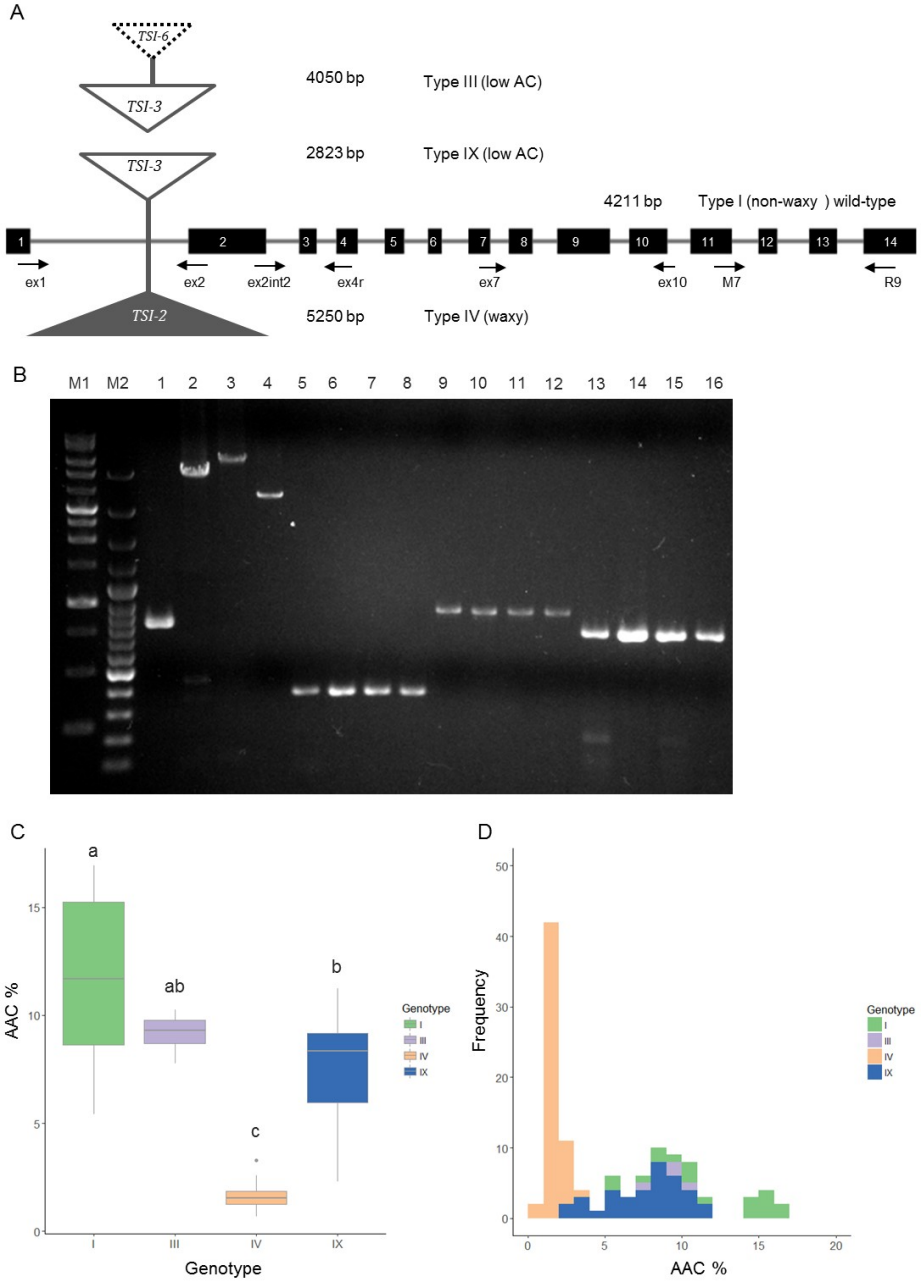
Assessment of *Wx* genotypes and phenotypes. (A) Schematic of the foxtail millet *Wx* gene. The positions of primer sets used to analyze genotypes are depicted, and primer sequences are listed in S2 Table. (B) PCR amplicons of foxtail millet *Wx* genotypes. Primer combinations, ex1 and ex2 (1–4), ex2int2 and ex4r (5–8), ex7 and ex10 (9–12), M7 and R9 (13–16), are indicated at the top of each the figure. M1 and M2 indicates 1 kb DNA ladder (DM3100 ExcelBand) and 100 bp DNA ladder (100 bp DNA ladder plus, GeneTeks BioScience Inc.), respectively. (C-D) The distribution of apparent amylose content (AAC) for each *Wx* genotype. A total of 114 accessions used for AAC assays were grown in the first crop season of 2015.

A total of 124 accessions were subjected to *Wx* genotype analysis, in comparison to the wild-type allele of 14 exons spanning 4,211 bp. Four *Wx* specific primer combinations, namely ex1/ex2, ext2int2/ex4r, M7/R9, and ex7/ex10 (S2 Table), were applied to identifying insertions within intron 1, the region from exon 2 to exon 4, intron 12, and exon 10 with wild-type amplicons of 828 bp, 379 bp, 759 bp, and 904 bp, respectively (Fig 1A and 1B). Only one of the four primer pairs, ex1/ex2, showed polymorphism with four alleles among accessions, indicating that TEs inserted in intron 1 rather than the other positions on *Wx* (Fig 1B). The amplicons showed that type I, III, IV, and IX alleles spanned 828 bp, 4 Kb, 5.2 Kb, and 2.8 Kb, respectively (Fig 1B). Positions of TE insertions in *Wx* were the same as published genotypes according to nucleotide BLAST (Fig 1A). Totals of 21, 5, 61, and 37 accessions were classified as type I, III, IV, and IX respectively (Fig 1B and S3 Table). Effects of four *Wx* genotypes on AAC were revealed in 114 accessions, with AC ranging from 0.69% to 16.92%. The AAC of type I ranged from 5.42% to 16.92% with an average of 11.91%; type III ranged from 7.78% to 10.25% with an average of 9.16%; type IV ranged from 0.69% to 3.29% with an average of 1.61%; type IX ranged from 2.32% to 11.25% with an average of 7.6% (Fig 1C and S3 Table). *Wx* could explain about 78% of the total variance in AAC. Three different genotypes, type I, IV, and IX, differed significantly in AAC, while type III had higher ACC than type IV but overlapped with types I and IX (Fig 1C and 1D and S3 Table). The majority of landraces in Taiwan had waxy endosperm (type IV), followed by low AC types (III and IX) (Fig 1D).

### Gene expression and protein contents of GBSSI in developing seeds

**Fig 2:**
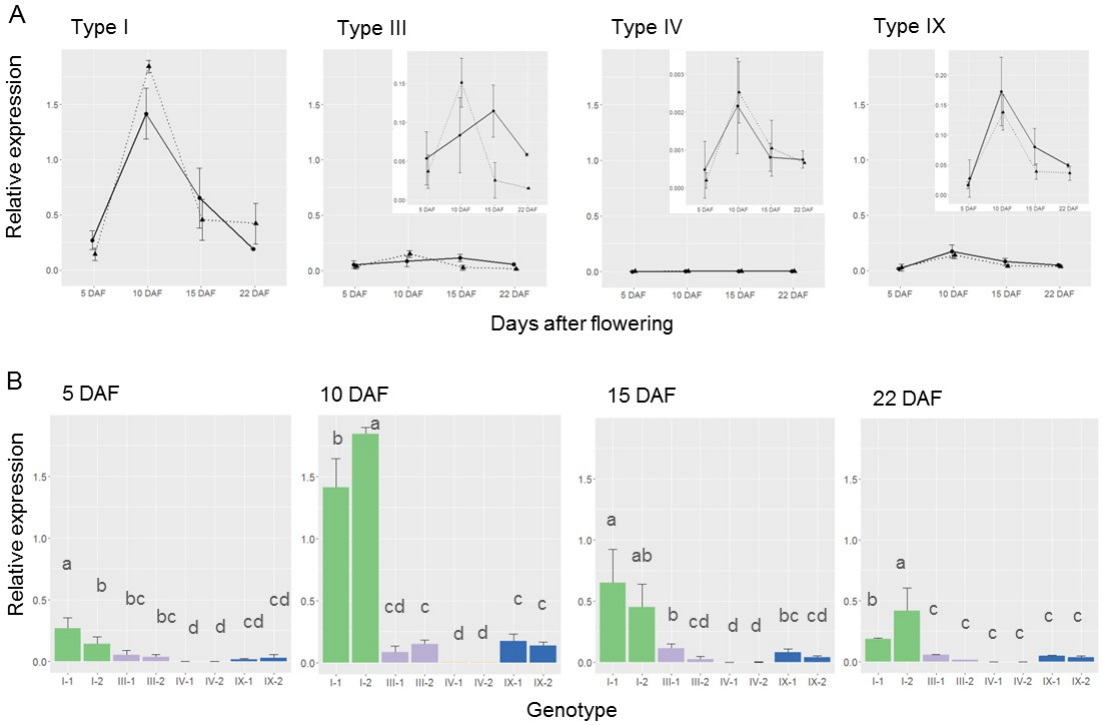
Gene expression of *Wx* and quantity of GBSSI protein for different *Wx* genotypes. (A) Expression levels of *Wx* at different seed development stages. The relative expression levels of four *Wx* genotypes, each represented by two accessions indicated by solid and dotted lines, were estimated at 5, 10, 15, and 22 DAF. Inserts on the right corners of figures of three mutated genotypes are the original expression patterns drawn without normalized scales. (B) Comparison of expression levels of *Wx* genotypes at different endosperm development stages. Genotype I, III, IV, and IX are represented by green, violet, orange (could be seen at 10 DAF, but not in other three stages), and blue, respectively. Means with different superscripts are significantly different at *p*<5%, according to Fisher’s LSD test.

Transcript levels of *Wx* were evaluated in two accessions for each of 4 *Wx* genotypes at 5, 10, 15, and 22 DAF during grain filling. In general, *Wx* expression levels peaked at 10 DAF and then gradually decreased (Fig 2A). At 5 DAF, there was no significant difference among the four genotypes. The wild-type, type I, had much higher *Wx* expression than the other three genotypes at later endosperm development stages, especially 10 DAF. Type IV, having the lowest expression level, was classified as a separate group at 10 DAF, while type III and type IX formed another. At 15 DAF, accession I-1 was much higher than the others but no notable difference was observed between types III and IX. However, at 22 DAF, the expression level of type I was significantly higher than the others. It is worth noting that the expression levels of accession I-2 at 10 and 22 DAF were significantly higher (LSD, *p*<0.05) than accession I-1 (Fig 2A and 2B).

**Fig 3:**
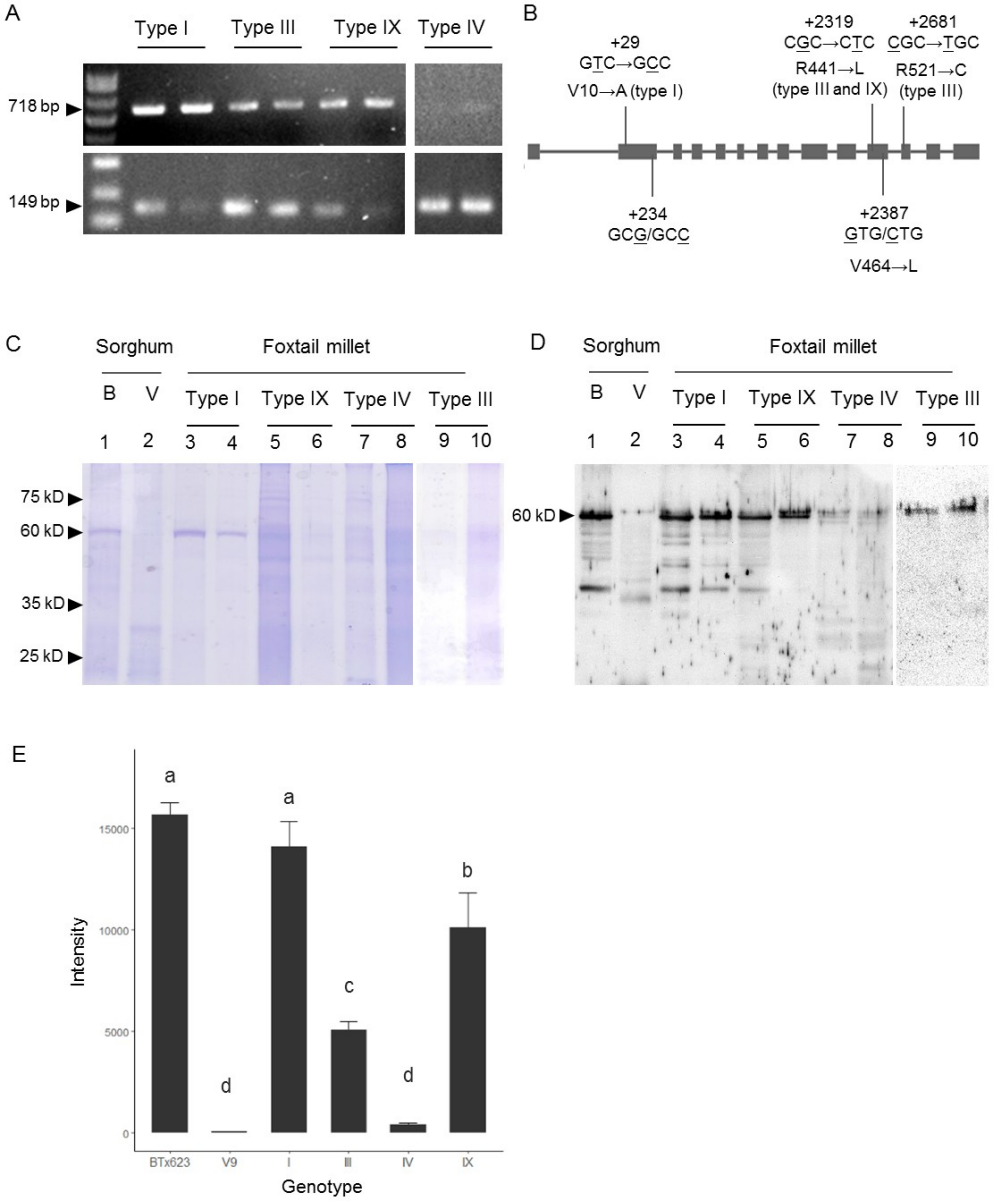
Genetic information for four *Wx* genotypes. (A) Transcripts of four *Wx* alleles in developing seeds at 10 DAF (upper panel). The housekeeping gene, *EF1a*, was used as internal control (lower panel). (B) SNP sites on different *Wx* genotypes. (C) Assay of granule-bound proteins of 2 sorghum and 8 foxtail millet accessions separated by SDS-PAGE. Lane 1: BTx623 (non-waxy sorghum); lane 2: V9 (waxy sorghum); Lanes 3 and 4: Type I (non-waxy type); lanes 5 and 6: Type IX (low AAC type); lanes 7 and 8: Type IV (waxy type); lanes 9 and 10: Type III (low AAC type). (D) Immunoblotting of GBSSI for different *Wx* genotypes. (E) GBSSI contents were reflected by band intensities of immunoblot quantified by ImageJ. Means with the different letter superscripts are significantly different at *p*<5% level, according to Fisher LSD test.

Only one kind of amplicon of mature mRNA was detected among the four genotypes, indicating that TE insertions did not cause alternative splicing (Fig 3A). Sequence analysis of cDNA derived from exon 2 to part of exon 14 that encompassed deduced amino acids from Met 1 to Pro 606 showed that all landraces from Taiwan shared high similarity with the reference variety, Yugu 1. Fig 3B depicted the observed variations compared to the reference sequence. Yugu 1, a non-waxy type from China, lacked an amino acid, Gly77, in comparison with Taiwanese accessions and other crops (Fig 3B and S1 Fig). All of the analyzed Taiwanese accessions had C at +234 and +2,387 rather than G, leading to synonymous substitution and a nonsynonymous substitution of V464L, respectively (Fig 3B). In addition, a SNP (C/T) at +29 was detected between two selected type I (non-waxy) accessions that changed the amino acid from V10A, while Yugu 1 possessed V. Another SNP (G/T) at +2319 causing R441L was found in both type III and type IX. Type III had an additional substitution (C/T) at +2681, leading to R521C. Except for the variation at the 10^th^ amino acid, the other three nonsynonymous mutations were located in the conserved domain of the *S*. *italica* GBSSI protein sequence, V82 to K572; however, they did not alter the highly conserved sequence that is commonly shared among cereals (S1 Fig).

Granule-bound proteins extracted from developing endosperm at 15 DAF of two accessions of each genotype were analyzed to determine whether differences in GBSSI protein amounts accounted for AC variation among four *Wx* genotypes. The granule-bound proteins with a molecular weight of ~60 kDa were detected with obvious SDS-PAGE bands in non-waxy sorghum, BTx623, and non-waxy foxtail millet (type I), but faint bands in waxy sorghum, V9, and low AC genotypes, III, IX, and IV (Fig 3C). The 60 kDa protein was then identified as GBSSI by immunoblot analysis using anti-SbWx (protein antiserum, 1/3,000), indicating that the amino acid sequences of foxtail millet and sorghum were highly similar (Fig 3D). Some proteins with molecular weight higher than 60 kDa were detected as faint bands on the SDS-PAGE, suggesting their relatively low concentration in starch granules compared to GBSSI. These proteins with molecular weight around 100 kDa or between 75 to 100 kDa might be some surface-associated proteins of starch granules such as SSIIa, SSI, SBEIIa, and/or SBEIIb (Fig 3C).

The GBSSI contents of non-waxy grains were significantly higher than those of waxy grains in both foxtail millet and sorghum. Among the four foxtail millet *Wx* genotypes, the three TE insertion genotypes had significantly lower protein quantities than wild-type in the following order: type IX > type III > type IV (Fig 3D and 3E). While type III and type IX were both classified as low AC, GBSSI protein quantities between these two types were significantly different at ~36% and ~72% of type I, respectively. In partial summary, GBSSI contents were clearly positively correlated with AAC in both foxtail millet and sorghum (Fig 3D and 3E).

### Genetic diversity of foxtail millet selected and preserved by indigenous peoples in Taiwan

**Fig 4:**
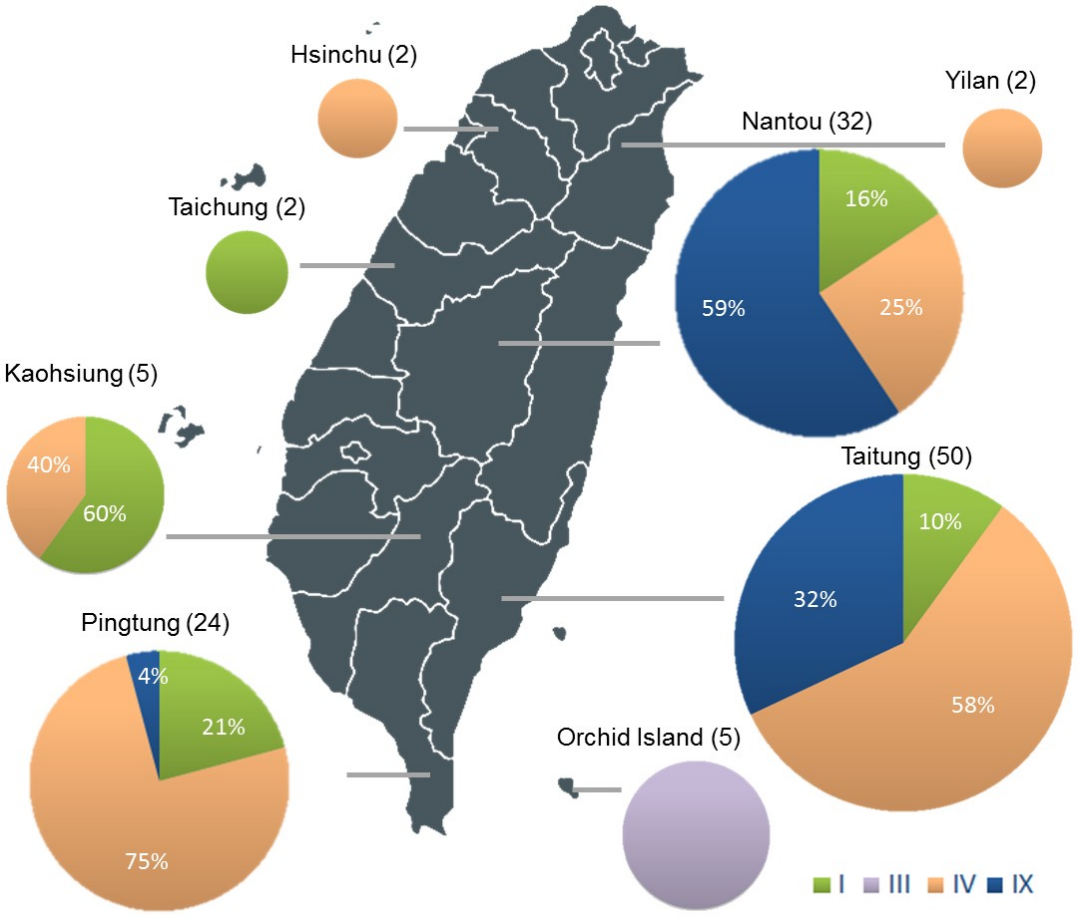
The geographical distribution of foxtail millet *Wx* genotypes in Taiwan. A total of 50 (40.3%), 32 (25.8%), 24 (19.4%), 5 (4%), 5 (4%), 2 (1.6%), 2 (1.6%), and 2 (1.6%) of 124 accessions were collected from Taitung, Nantou, Pingtung, Orchid Island, Kaohsiung, Yilan, Taichung, and Hsinchu, respectively. The map was adopted from https://simplemaps.com/resources/svg-tw.

Foxtail millet is a traditional staple food for indigenous peoples in Taiwan, along with taro, Upland rice, and sweet potato. In addition, foxtail millet is perceived as a sacred crop that is used ceremonially by indigenous peoples. Currently, 16 ethnic groups of Austronesian Taiwanese are recognized, mainly distributed over much of the Central Mountain Range of the main island. Most foxtail millet accessions were collected from Taitung County, followed by Nantou and Pingtung Counties with 50, 32, and 24 accessions, respectively (Fig 4). Traditional uses of foxtail millet of five ethnic groups from seven regions were investigated, finding that different tribes belonging to the same ethnic group often have common uses (Table 1). Atayal people use cooked foxtail millet for lactate produced during starch fermentation to preserve meat or fish by pickling with salt. Bunun people in Taitung and Nantou Counties use foxtail millet for brewing, porridge, ancestral worship, gifts and decoration. Rukai and Paiwan peoples share common applications, including foxtail millet wine, seasoning for pickling meat and fish, porridge (*lrubu* in Rukai), dumplings and steamed sticky cakes, ancestral worship and gifts. Rukai people also use foxtail millet for divination. Foxtail millet dumplings, *cinabu* and *abai* of Paiwan of Rukai, are traditional delicacies served only during rituals and harvest festivals but are now available in authentic restaurants anytime (Table 1). *Cinabu* or *abai* is mainly made from dehusked or polished grains of waxy varieties with glutinous endosperm, whereas porridge is made from non-waxy or low AC grains. Notably, instead of cultivating foxtail millet for making foods or brewing wine, Tao people only use foxtail millet for ancestral worship and gifts, which have much to do with shapes and colors of panicles instead of grain quality. Landraces were preserved by different ethnic groups owing to various applications under different food processing methods and cultures.

The *Wx* genotypes of foxtail millet landraces preserved and cultivated by indigenous peoples in Taiwan might reflect selection for different preferences. In general, glutinous foxtail millet is the most popular type for making traditional foods and wine. Sixty-one of 124 accessions (~49.2%) were identified as waxy (type IV), and were prevalent in southern Taiwan (Fig 4). *Wx* genotypes identified in Taiwan had different geographic distributions, which were related to various ethnic groups. In the 32 accessions of Nantou County where Ataya and Bunun tribes are located, 16%, 25%, and 59% of the accessions were classified as type I (non-waxy), type IV (waxy), and type IX (low AC), respectively. Twenty-four accessions collected from Pingtung County where the Rukai and Paiwan peoples are distributed consist of 21%, 75%, and 4% type I, type IV and type IX, respectively. Among 50 accessions collected from Taitung County where Bunun and Paiwan peoples are distributed, type I, type IV, and type IX accounted for 10%, 58%, and 32% of the total, respectively. Five accessions classified as type III were all collected from Orchid Island, a small island about 90 km away from Taiwan where the Tao people live, who do not use foxtail millet as staple food.

## Discussion

Foxtail millet has become a preferred choice for addressing food and nutritional security in the semi-arid tropics because of its relatively short life cycle, abundant seed production, drought tolerance, and high nutritional value. Because of global climate change, foxtail and other millets, being water-efficient cereals, are proposed to replace rice for water savings and production of essential nutrients. Foxtail millet has been the only millet cultivated throughout Taiwan and a symbolic food served in festivals and rituals of indigenous peoples for more than 5,000 years. Since food products that deliver taste, color and texture with stable quality is the ultimate goal for which foxtail millet is utilized, grain quality is a major concern. In this study, AC and functional study of *Wx* alleles of 124 accessions were evaluated. Landraces were preserved because of indigenous preferences, contributing to the high diversity of foxtail millet collections in Taiwan reflected by four *Wx* genotypes with widely ranging AC (Fig 1). Three mutated *Wx* alleles resulting from TE insertions reduce *Wx* expression and decrease GBSSI protein content, leading to low AC in the endosperm (Figs 2 and 3). The geographical distributions of *Wx* genotypes were associated with selection for specific applications by Taiwanese indigenous people (Table 1 and Fig 4).

### Genetic diversity of amylose content in Taiwanese foxtail millet landraces

Assessing the genetic diversity of Taiwanese foxtail millet germplasm is important for the conservation of genetic resources and practical applications, including advancing grain quality to meet diverse demands. Four genotypes found in this study, type I, III, IV, IX, were consistent with previous reports about collections from Taiwan (Fig 1) [38]. Types IV and IX were waxy and low-AC, due to insertions of two *En/Spm-like* transposons, *TSI-2* with 5,250 bp and *TSI-3* with 2,823 bp, respectively. Type III, a low AC type, had additional insertion of a putative non-autonomous transposon *TSI-6*, with 1,226 bp in *TSI-3*. Type I is widely distributed throughout Eurasia followed by type IV among 13 *Wx* genotypes [38]. The *Wx* genotypes from East and Southeast Asia were rich in various low-AC and waxy types in comparison with those from Europe, Central Asia, and South Asia, that mainly possessed type I. The AC (5.42–16.92%) of the non-waxy types in Taiwanese landraces were generally lower than those of the same types from other regions such as East Asia, South Asia, and Western countries (S3 Table) [39]. Taiwan has a relative abundance of landraces possessing type IV in comparison to other regions across Eurasia (Fig 4 and S2 Fig). The majority of landraces in Taitung and Pingtung were type IV, with waxy grains, while type IX (low AC) was the majority in Nantou (Fig 4). Notably, all five accessions identified as type III (low AC) were from Orchid Island, which is a small island situated off the southeastern coast of Taiwan.

Glutinous foxtail millet is favored by indigenous peoples for its physicochemical properties, which play an important role in making various kinds of foods and wine. Kida (1941) reported that about half of the local varieties collected from all over Taiwan in the 1930’s was glutinous. In particular, glutinous varieties that possessed type IV allele were prevalent in southern Taiwan where Paiwan and Rukai peoples mainly distributed, while none were found in Batan Islands in the Philippines [47, 48]. Foxtail millet dumplings, *cinabu or cinavu*, are grains of steamed and flavored foxtail millet wrapped with Macaranga or Khasya trichodesma leaves, which originated from Rukai and Paiwan peoples. A sticky cake is a general term representing several kinds of foods made from glutinous foxtail millet or rice. For example, Atayal people distributed in Nantou County makes sticky cake called *hekkel* by pounding steamed foxtail millet grain with a mortar and pestle until sticky paste forms. Another type of sticky cake, *abai*, is originated from Rukai and Paiwan peoples and its ingredients are similar to foxtail millet dumplings except dough is used rather than grains. In addition, in most of the ethnic groups except Tao, millet wine is ubiquitous, consumed daily and during ancestral worship as well. Therefore, these landraces were presumably preserved by indigenous people due to their preference for sticky properties in daily foods and beverages (Table 1). Bunun people distributed in Nantou and Taitung counties prefer foxtail millet with low-AC endosperm for daily meals such as porridge, while the waxy type cultivated in a relatively small portion was mainly used for wine making. Interestingly, people have had a particular preference for glutinous cereals in East Asia. In addition to foxtail millet, waxy grains are very popular in landraces of several cereal crops, such as rice, maize, sorghum, barley, common millet, and Job’s tears (*Coix lacryma-jobi*). For example, Job’s tears is mainly cultivated in East Asia (e.g., Japan, Korea, and China), Southeast and South Asia for food, medicine and fodder. Many Job’s tears cultivars have waxy endosperm derived from its non-waxy wild ancestor, *C*. *lacryma-jobi* var. *lacryma jobi* L. [35]. Coincidently, waxy landraces of several minor cereal crops had been selected based on human preferences for sticky food and brewing, even more for essential oblation.

### The relationship between *Wx* genotypes and amylose content

A total of thirteen *Wx* alleles caused by various TE insertions were identified, leading to non-waxy, low-AC, and waxy endosperm types [38–40]. In this study of 124 accessions, four genotypes, type I, III, IV, and IX, were identified and accounted for 78% of AC variation in the range of 0.69–16.92%. Types III, IV and IX resulted from insertions of *TSI-3* together with *TSI-6*, *TSI-2*, and *TSI-3*, with insertion sizes of 4,050 bp, 5,250 bp, and 2,823 bp in intron 1, respectively (Fig 1A). TE insertions of *Wx*, which lead to alternative splicing resulting in several transcripts, were found in maize [7, 26]. However, these three TE insertions into *Wx* in foxtail millet affected splicing efficiency rather than splicing accuracy, leading to low transcription levels of the same transcripts (Figs 2 and 3A). The gene expression profiles during four grain filling stages demonstrated similar trends among these four genotypes, peaking at 10 DAF then declining, but nonetheless differing from each other significantly during the grain filling stages (Fig 2A). At the peak stage, 10 DAF, *Wx* expression of type I was ~12, ~695, and ~11 fold higher than types III, IV, and IX, respectively (Fig 2B). Type IV, with the largest insertion, exhibited the lowest gene expression through grain filling stages, suggesting that its larger TE insertion had greater impact on splicing efficiency. The AAC of I-1 and I-2 were 14.35% and 15.26%, respectively, which might result from the difference in expression levels of these two accessions. However, in the other three mutated genotypes, there were no significant differences among the selected accessions.

In many plant species, starch biosynthetic enzymes can be divided into two categories, soluble and granule-associated [49]. The other group consists of internal granule-associated proteins that require gelatinization by boiling the starch in a buffer with SDS [49]. The molecular weight of GBSSI protein isolated from the immature endosperm of foxtail millet was ~60 kDa, while it was predicted to be ~66 kDa by using the Protein Molecular Weight Calculator (http://www.sciencegateway.org/tools/proteinmw.htm) (Fig 3D). This observation was consistent with other cereal crops, such as sorghum [34]. Additionally, the protein molecular weight of GBSSI in maize, rice, barley, wheat and dicot species were reported to be ~60 kDa [45, 49]. GBSSI protein sequences among different cereal crops were conserved and the sequences of sorghum and foxtail millet shared high similarity; thus, the antiserum of sorghum GBSSI could be applied to measure the amount of GBSSI in four different *Wx* genotypes of foxtail millet (Fig 3D).

Physicochemical properties reflected by the ratio of amylose and amylopectin are indices for starch utilization in various food and industrial applications. The proportions of amylose and amylopectin in cereal grains are regulated by starch synthesis genes, branching and debranching enzyme genes, and starch synthesis-related genes. *Wx* is the key gene to determine the GBSSI enzyme amount and activity, and consequently AC. This study showed that wild-type *Wx* alleles of sorghum and foxtail millet had the highest amounts of GBSSI and AC. In contrast, type IV, one of the mutated alleles, had the least GBSSI and lowest AC, whiles type III and IX had intermediate GBSSI and low AC between wild-type and type IV (Fig 3D and 3E). The low-AC accessions were capable of forming GBSSI but the decreased amount of protein led to relatively low AC in comparison with non-waxy types. However, rather than the absence of GBSSI protein mentioned in previous studies, waxy accessions still generate functional GBSSI even though its content was quite low (Fig 3E) [39]. In the diversity panel of 124 accessions, the AC ranges of type III, IV, and IX were significantly lower than type I. Variation of AC within the same genotypes might be caused by environmental effects, other starch synthesis related genes and interactions among genes and environment. Nevertheless, SNPs resulting in non-synonymous mutations could not be ruled out. One non-synonymous substitution that altered V 10 to A was uncovered within type I. Non-synonymous substitutions, R441L in type III and IX, and R521C in type III, were also observed among different types (Fig 3B). In comparison with Yugu 1, all accessions from Taiwan had C rather than G at +234 and +2,387, which turned out to be synonymous and nonsynonymous substitutions, respectively. These SNPs might reduce enzyme activity to alter AC, a phenomenon that was very common in other cereal crops [50, 51]. The *Wx* of rice is the best-studied case. Some rice waxy alleles were chemically induced mutant, such as *Wx*^*mq*^ and *WY1* [22, 23]. However, several naturally occurring alleles in landraces *Wx*^*op*^, *Wx*^*hp*^, and *Wx*^*zm*^ possessing low AC because of nonsynonymous substitutions, were identified and preserved in Yunnan landraces [20, 21, 52].

### Geographical distribution of *Wx* genotypes associated with human behavior

Three mutated *Wx* alleles were identified in Taiwanese foxtail millet landraces, with type IV and IX inserted by *TSI-2* and *TSI-3*, respectively, while type III had *TSI-6* nested in *TSI-3* (Fig 1). Of these, *TSI-2* and *TSI-3* were both classified as autonomous *En*/*Spm*-like transposons, whereas *TSI-6* was non-autonomous [38]. Most of these mutated *Wx* alleles occurred by TE insertions, which were also found in maize, sorghum, and wheat but not rice [20, 26, 34]. In foxtail millet, *Wx* was inserted by both DNA transposon and retrotransposon, which were followed by nested insertions, deletion, and/or intragenic recombination, indicating that TE were relatively active in some period during thousands of years of human selection [38].

A total of 13 *Wx* alleles in foxtail millet were identified by PCR-based methods from 871 accessions collected from several countries through Eurasia [38]. The non-waxy wild-type (type I) is widely distributed throughout Eurasia as well as its wild ancestor, green foxtail [17, 38, 40, 53]. Non-waxy landraces from South Asia and countries to the west showed slightly higher amylose content than those from East and Southeast Asia [39]. Except type I, the wild-type, which was prevalent all over the world, the mutated *wx* alleles displayed specific distributions. Type IV, the waxy allele, was found in Taiwan, and also in China, Thailand, India, Myanmar, Indonesia, Korea, and Japan (S2 Fig). Type IX, the low-AC allele, had a distribution limited to Taiwan, the Philippines, and India. Although there was only an individual identified as type IX among 43 accessions from Taiwan in the previous study, type IX occupied nearly 30% of analyzed germplasm in this study mainly distributed in Nantou County, where most Bunun people live (Fig 4). Bunun people favored foxtail millets with slightly sticky endosperm called “malutas”, thus type IX was selected and cultivated in Bunun tribes [54] TypeIII, the low-AC allele, only occurred in a very restricted area, namely, the Philippines, Taiwan and Japan (S2 Fig). Waxy and low-AC grains were relatively popular in Taiwan as well as in Korea and Japan, while non-waxy grains were dominant in other regions.

Type III was also found in the Nansei Islands of Japan with 2 accessions and the Philippines with 5 accessions, and Taiwan with 6 accessions. Five out of 6 accessions came from Orchid Island and the rest came from Pingtung County (S2 Fig) [38]. Among the landraces collected from Orchid Island, 3 of 4 accessions were classified as low-AC along with one waxy accession, and 2 of 3 accessions were type III with low AC and the other one was waxy [39, 40]. In the present study, however, all 5 accessions identified as type III are from Orchid Island. The uniqueness of the distribution of type III in Orchid Island, which is located 90 kilometers from the southeast coast of Taiwan in the Pacific Ocean, was assumed to be associated with the agricultural tradition and custom of the people indigenous to Orchid Island, the Tao. The Tao are the only non-Formosan Austronesian speakers among Taiwanese indigenous peoples [55]. Tao people traditionally rely heavily on fishing for survival, growing taro as a primary staple food, and they do not brew millet wine (Table 1). Thus, foxtail millet, a minor crop for the Tao, had been selected and cultivated for different purposes in Orchid Island, as compared to Taiwan Island.

Foxtail millet accessions collected from Taiwan clustered with those from the Nansei Islands and the Philippines, based on genetic relationships determined by transposon display (TD) of *Wx* alleles [56]. Two accessions of type III found in Japan came from Ikema and Ishigaki islands, which are closer to Taiwan than other islands among the Nansei such as Okinawa [38]. No waxy landrace was found in Batanes in the Philippines (Fig 4). The Ivatan islanders are inhabitants of Itbayat in the Batanes, which is an archipelago located between Taiwan and the Philippines. The languages of the Tao and Ivatan belong to the 10^th^ branch of the Austronesian language group, and these two populations share cultural similarities [57, 58]. Northward gene flow from the Philippines to Orchid Island seemed to be independent of cultural interflow; in other words, trading resulted in linguistic affinity among Tao, Ivatan, and the Philippines, but had small impacts on genetic exchanges [59]. Type III foxtail millet found in the Nansei islands might have been introduced by Neolithic trading. Thus, the spread of this type might reflect the migration of Tao and their relatives.

Foxtail millet, a symbolic crop for indigenous people in Taiwan, has been cultivated for more than 5,000 years [15]. Since different ethnic peoples have their own cultures, foxtail millet has been applied in various food processing and worship practices (Table 1). The distribution of 16 ethnic groups is mainly over much of the Central Mountain Range of the island. Hence, languages and cultures among different ethnic groups were relatively isolated and quite different from each other, with rare interactions and migrations between groups. Different preferred selections and little seed exchange might have contributed to the high genetic diversity of foxtail millet in Taiwan. The distribution of different genotypes was not even, for example type III only being found in Orchid Island, and type IX mainly distributed in Nantou County. However, type IV was commonly distributed within Taiwan island since glutinous grain was needed in brewing, festivals, and ancestor worship. Nevertheless, genetic drift due to small amounts of preserved seeds increasing genetic diversity could not be neglected.

Taiwan is the northernmost point of the distribution of the Austronesian people and is believed to be the origin of their expansion in prehistory. Orchid Island is located along the east coast of Taiwan between Ikema and Ishigaki islands in Japan, and Batanes in the Philippines, where type III accessions were also observed in the previous study [38]. In fact, archeological artifacts found in the eastern coast of Taiwan were also found on Orchid Island and Batanes, supporting the “Out of Taiwan” hypothesis of Austronesian expansion, indicating that settlement on the islands might date back to 4,000 YBP [59]. Several pieces of evidence also suggest that Taiwan is where the languages and cultures of the Austronesians originated thousands of years ago, which include people in the Pacific Islands, Southeast Asia, the Maoris in New Zealand and Polynesians in Hawaii. Furthermore, the phylogeographic pattern of paper mulberry by using chloroplast DNA supported the “Out of Taiwan” hypothesis [60]. The type III *Wx* genotype which is unique to landraces Orchid island might be due to the interactions between Taiwanese Austronesian and Orchid Islanders have ceased much earlier than those between Orchid islanders and the Batanes archipelago islanders [59]. Further studies using phylogenomics of foxtail millet in East and Southeast Asia may unveil further insights into prehistoric and historic human movements in the Pacific.

## Conclusions

*Wx* alleles caused by the presence of different TEs resulted in reduced *Wx* mRNA by undermining transcription efficiency, leading to lower levels of GBSSI protein content and reduced AC. Geographic distribution of *Wx* genotypes seemed to be associated with preferences for specific endosperm types under human selection and through migration. The evidence that type III from Taiwan was limited to the Orchid Island strongly supports the hypothesis that foxtail millet distribution, human migration and cultural interflow of indigenous people had a close relationship. This study provides new evidence for how *Wx* alleles affect AC and revealed the relationship between the diversity of Taiwanese foxtail millet germplasm and traditional applications in aboriginal tribes throughout Taiwan’s history.

## Supporting information

S1 Fig Alignment of amino acid sequences of GBSSI of 15 crop species. Predicted amino acid sequences of GBSSI from monocot and dicot crops were aligned using ClustalW (MEGA version 7). The alignment includes the amino acid residues around the SNPs detected among four *Wx* genotypes of foxtail millet, which is denoted by the asterisks, and the highly conserved amino acids are highlighted in yellow.
(PDF)

S2 Fig Geographical distribution of foxtail millet in Asia, Europe, and Africa. Genotypes of Taiwan’s foxtail millet were obtained from this study, and the others were adapted from Kawase et al. (2005). Numbers in parenthesis denotes the number of accessions collected from the country. The map was adapted from FreeVectorMaps.com.
(PDF)

S1 Table The list of foxtail millet accessions studied by accession, PI number, origin, PCR analysis, and the apparent amylose content. (DOCX)

S2 Table Primers used to assess *Wx* genotypes. (DOCX)

S3 Table List of foxtail millet accessions studied by accession, *Wx* allele, TE insertion, and apparent amylose content. (DOCX)
