# Supplementary material for: Waxy allele diversification in foxtail millet (Setaria italica) landraces of Taiwan
Source: PLoS One. 2018 Dec 31;13(12):e0210025. doi: 10.1371/journal.pone.0210025 (PMC6312202; doi:10.1371/journal.pone.0210025)
Supplement: S1 Fig — Predicted amino acid sequences of GBSSI from monocot and dicot crops were aligned using ClustalW (MEGA version 7). The alignment includes the amino acid residues around the SNPs detected among four Wx genotypes of foxtail millet, which is denoted by the asterisks, and the highly conserved amino acids are highlighted in yellow. (PDF) [file pone.0210025.s001.pdf]

										*										*			
NP 001267750.1 Setaria italica	M	A	A	L	A	T	S	Q	L	V	~	69	P	S	L	V	V	C	A	T	-	-	A	G
XP 021603997.1 Manihot esculenta	M	A	T	V	I	A	A	H	-	L	~	72	P	-	-	A	A	K	I	I	C	G	H	G
XP 015644489.1 Oryza sativa Japonica Group	M	S	A	L	T	T	S	Q	L	A	~	72	P	S	V	V	V	Y	A	T	G	-	A	G
XP 002436418.1 Sorghum bicolor	M	S	T	L	A	T	S	Q	L	V	~	72	P	S	L	V	V	C	A	T	-	-	A	G
XP 006343762.1 Solanum tuberosum	M	A	S	I	T	A	S	H	H	F	~	69	P	G	C	S	A	T	I	V	C	G	K	G
NP 001237971.1 Glycine max	M	A	T	V	T	A	S	S	Y	A	~	72	D	G	V	L	G	R	-	-	I	K	C	G
NP 001105001.3 Zea mays	M	A	A	L	A	T	S	Q	L	V	~	70	P	S	L	V	V	C	A	S	-	-	A	G
CAC69955.1 Pisum sativum	M	A	T	V	T	A	S	S	N	I	~	75	K	N	V	R	P	K	G	I	I	V	C	G
BAM38098.1 Echinochloa esculenta	M	A	A	L	A	T	S	Q	L	V	~	71	P	S	L	V	V	C	A	T	G	-	A	G
BAI44033.1 Amaranthus hybridus	M	E	T	V	T	S	S	H	-	F	~	74	P	-	-	-	-	-	-	F	I	R	S	G
BAA82346.1 Phaseolus vulgaris	M	A	T	V	S	M	A	S	C	V	~	70	E	K	V	L	E	K	-	-	I	E	C	G
ADA61154.1 Panicum miliaceum	-	-	-	-	-	-	-	-	-	-	~		-	-	-	-	-	-	-	-	A	-	A	G
AAQ06271.1 Pennisetum glaucum	M	A	A	L	A	T	S	Q	L	A	~	72	P	S	L	V	V	C	A	T	G	-	A	G
AAM74049.1 Hordeum vulgare	M	A	A	L	A	T	S	Q	L	A	~	70	L	S	V	V	V	R	A	T	G	-	S	G
AAG27624.1 Triticum aestivum	M	A	A	L	V	T	S	Q	L	A	~	66	L	S	M	V	V	R	A	T	G	S	A	G

										*											*		
NP 001267750.1 Setaria italica (432)	L	G	T	G	K	K	K	F	E	R	~	454	R	A	V	V	K	F	N	A	A	V	A	H
XP 021603997.1 Manihot esculenta (434)	L	G	T	G	K	K	K	F	E	K	~	456	R	G	V	A	K	F	N	V	P	L	A	H
XP 015644489.1 Oryza sativa Japonica Group (437)	L	G	T	G	K	K	K	F	E	K	~	459	R	A	V	V	K	F	N	A	P	L	A	H
XP 002436418.1 Sorghum bicolor (434)	L	G	T	G	K	K	K	F	E	R	~	456	R	A	V	V	K	F	N	A	A	L	A	H
XP 006343762.1 Solanum tuberosum (461)	L	G	T	G	K	K	E	F	E	Q	~	483	K	G	V	A	K	F	N	V	P	L	A	H
NP 001237971.1 Glycine max (434)	L	G	T	G	K	K	I	M	E	K	~	456	R	G	V	A	K	F	N	G	P	L	A	H
NP 001105001.3 Zea mays (435)	L	G	T	G	K	K	K	F	E	R	~	457	R	A	V	V	K	F	N	A	A	L	A	H
CAC69955.1 Pisum sativum (439)	L	G	T	G	K	K	E	M	E	K	~	461	R	G	V	A	K	F	N	V	P	L	A	H
BAM38098.1 Echinochloa esculenta (434)	L	G	T	G	K	K	K	F	E	K	~	456	R	A	V	V	K	F	N	A	A	M	A	H
BAI44033.1 Amaranthus hybridus (432)	L	G	T	G	K	E	V	M	E	K	~	454	R	G	V	T	K	F	N	S	P	L	A	H
BAA82346.1 Phaseolus vulgaris (432)	L	G	T	G	K	K	S	M	E	K	~	454	R	G	I	A	K	F	D	G	P	L	A	H
ADA61154.1 Panicum miliaceum	L	G	T	G	K	K	K	F	E	R	~		R	A	V	V	K	F	N	A	A	L	A	H
AAQ06271.1 Pennisetum glaucum (435)	L	G	T	G	K	K	K	F	E	R	~	457	R	A	V	V	K	F	N	A	A	L	A	H
AAM74049.1 Hordeum vulgare (434)	L	G	T	G	K	K	K	F	E	K	~	456	R	A	V	V	R	F	N	A	P	L	A	H
AAG27624.1 Triticum aestivum (431)	L	G	T	G	K	K	K	F	E	R	~	453	R	A	V	V	R	F	N	A	P	L	A	H

				*																			
NP 001267750.1 Setaria italica (517)	H	M	G	R	L	S	V	D	C	K	V	V	E	P	A	D	V	Q	K	V	A	S	T	L
XP 021603997.1 Manihot esculenta (519)	Q	M	G	A	L	R	V	E	C	D	K	I	D	S	A	D	V	A	A	I	V	K	T	V
XP 015644489.1 Oryza sativa Japonica Group (522)	H	M	G	R	L	S	V	D	C	K	V	V	E	P	S	D	V	K	K	V	A	A	T	L
XP 002436418.1 Sorghum bicolor (519)	H	M	G	R	L	S	V	D	C	N	V	V	E	P	A	D	V	K	K	V	A	T	T	L
XP 006343762.1 Solanum tuberosum (546)	H	M	G	A	F	N	V	E	C	D	V	V	D	P	A	D	V	L	K	I	V	T	T	V
NP 001237971.1 Glycine max (519)	H	M	G	A	F	N	V	E	C	E	A	V	D	P	V	D	V	E	K	L	A	T	T	V
NP 001105001.3 Zea mays (520)	H	M	G	R	L	S	V	D	C	N	V	V	E	P	A	D	V	K	K	V	A	T	T	L
CAC69955.1 Pisum sativum (524)	H	M	G	S	F	N	V	K	C	D	A	V	D	P	V	D	V	D	A	I	P	K	T	V
BAM38098.1 Echinochloa esculenta (519)	H	M	G	R	L	S	V	D	C	K	V	V	E	P	A	D	V	Q	K	V	A	S	T	L
BAI44033.1 Amaranthus hybridus (517)	H	M	G	R	F	S	A	N	C	D	M	V	D	P	A	D	I	S	A	V	E	T	T	V
BAA82346.1 Phaseolus vulgaris (517)	H	M	G	A	F	N	V	D	C	E	A	I	D	P	A	D	V	E	K	I	A	T	T	V
ADA61154.1 Panicum miliaceum	H	T	G	R	L	S	V	D	C	K	V	V	E	P	A	D	V	Q	K	V	A	T	T	L
AAQ06271.1 Pennisetum glaucum (520)	H	M	G	R	L	S	V	D	C	K	V	V	E	P	A	D	V	Q	K	V	A	S	T	L
AAM74049.1 Hordeum vulgare (519)	H	M	G	R	L	S	V	D	C	N	V	V	E	P	A	D	V	K	K	V	A	T	T	L
AAG27624.1 Triticum aestivum (516)	H	M	G	H	L	S	V	D	C	N	V	V	E	P	A	D	V	K	K	V	V	T	T	L
