# Supplementary figures and images for: Waxy allele diversification in foxtail millet (Setaria italica) landraces of Taiwan
Source: PLoS One. 2018 Dec 31;13(12):e0210025. doi: 10.1371/journal.pone.0210025 (PMC6312202; doi:10.1371/journal.pone.0210025)

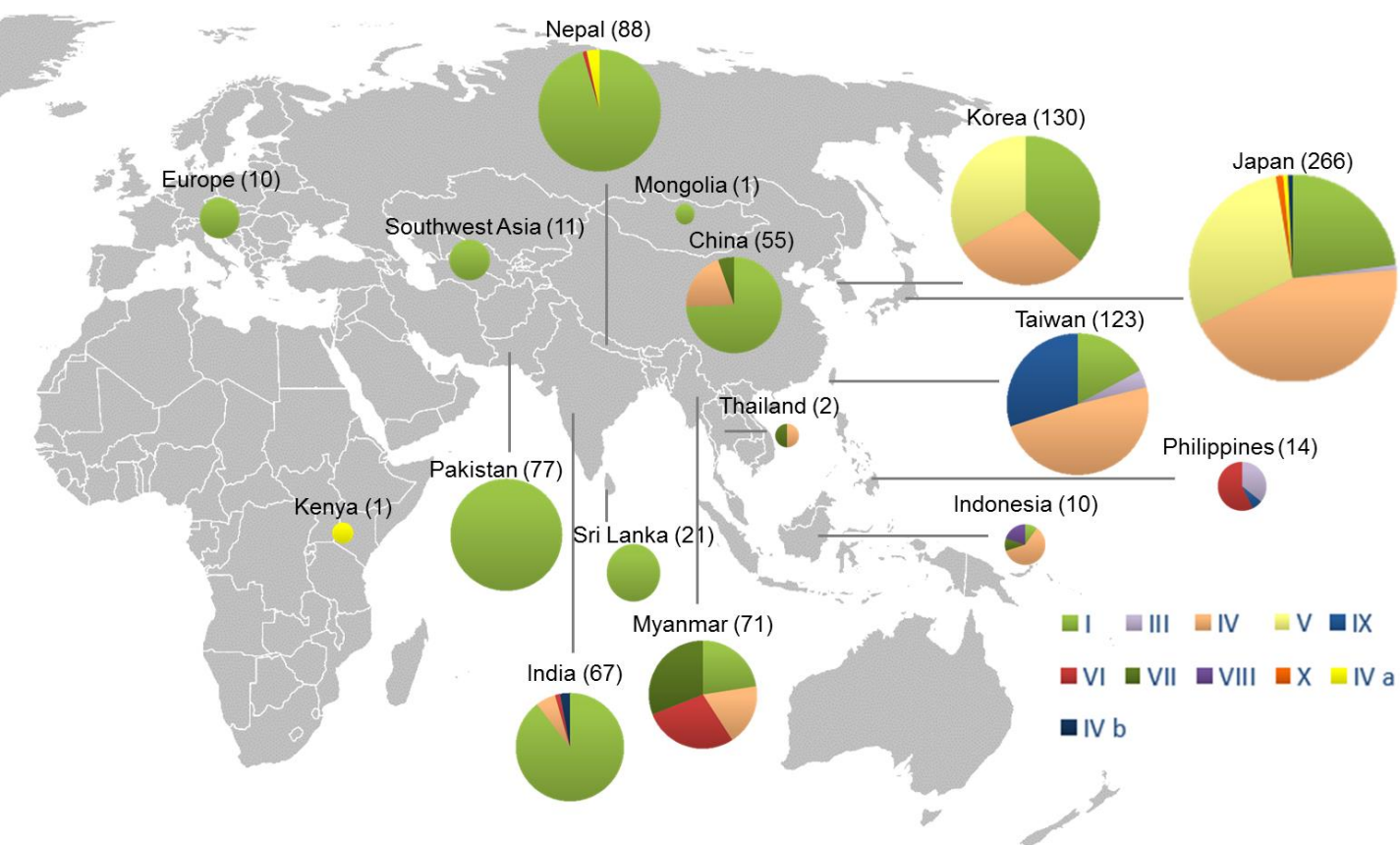

Supplement: S2 Fig — Genotypes of Taiwan’s foxtail millet were obtained from this study, and the others were adapted from Kawase et al. (2005). Numbers in parenthesis denotes the number of accessions collected from the country. The map was adapted from FreeVectorMaps.com. (PDF) [file pone.0210025.s002.pdf]
