# Supplementary material for: Waxy allele diversification in foxtail millet (Setaria italica) landraces of Taiwan
Source: PLoS One. 2018 Dec 31;13(12):e0210025. doi: 10.1371/journal.pone.0210025 (PMC6312202; doi:10.1371/journal.pone.0210025)
Supplement: S1 Table — (DOCX) [file pone.0210025.s003.docx]

## **S1 Table. The list of foxtail millet accessions studied by PI number, origin, PCR analysis and the apparent amylose content (AAC).**

| Accession | PI number | Origin ^a^ | Coordinates ^b^ | PCR amplification ^c^ | | | | AAC (%) ^d^ |
| --- | --- | --- | --- | --- | --- | --- | --- | --- |
|  |  |  |  | ex1/ex2 | ex2int2/ex4r | M5/R9 | M7/R9 |  |
| 382 | PI433382 | TTDARES, Taitung | 121E09' 22N45' | IV | a | a | a | N. A. |
| 383 | PI433383 | TTDARES, Taitung | 121E09' 22N45' | IV | a | a | a | 1.57 |
| 384 | PI433384 | TTDARES, Taitung | 121E09' 22N45' | IV | a | a | a | 1.27 |
| 385 | PI433385 | TTDARES, Taitung | 121E09’ 22N45' | IV | a | a | a | 11.70 |
| 386 | PI433386 | TTDARES, Taitung | 121E09' 22N45' | IV | a | a | a | 1.45 |
| 387 | PI433387 | TDAIS, Changhua | 120E32' 24N00' | I | a | a | a | 14.35 |
| 388 | PI433388 | Wutai, Pingtung | 120E43' 22N45' | IV | a | a | a | 1.26 |
| 389 | PI433389 | Wutai, Pingtung | 120E43' 22N45' | IV | a | a | a | 1.69 |
| 390 | PI433390 | Wutai, Pingtung | 120E43' 22N45' | IV | a | a | a | 1.62 |
| 391 | PI433391 | Wutai, Pingtung | 120E43' 22N45' | IX | a | a | a | 3.20 |
| 392 | PI433392 | Wutai, Pingtung | 120E43' 22N45' | IV | a | a | a | 2.56 |
| 393 | PI433393 | Wutai, Pingtung | 120E43' 22N45' | IV | a | a | a | 1.08 |
| 394 | PI433394 | Wutai, Pingtung | 120E43' 22N45' | IV | a | a | a | 1.01 |
| 395 | PI433395 | Wutai, Pingtung | 120E43' 22N45' | IV | a | a | a | 1.06 |
| 396 | PI433396 | Wutai, Pingtung | 120E43' 22N45' | IV | a | a | a | 1.10 |
| 397 | PI433397 | Wutai, Pingtung | 120E43' 22N45' | I | a | a | a | 15.83 |
| 398 | PI433398 | Wutai, Pingtung | 120E43' 22N45' | I | a | a | a | 5.42 |
| 399 | PI433399 | Shinyi, Nantou | 120E51' 23N42' | IX | a | a | a | 9.16 |
| 400 | PI433400 | Shinyi, Nantou | 120E51' 23N42' | I | a | a | a | 7.98 |
| 401 | PI433401 | Shinyi, Nantou | 120E51' 23N42' | IX | a | a | a | 8.44 |
| 402 | PI433402 | Shinyi, Nantou | 120E51' 23N42' | IX | a | a | a | 6.60 |
| 403 | PI433403 | Shinyi, Nantou | 120E51' 23N42' | IX | a | a | a | 9.27 |
| 405 | PI433405 | Shinyi, Nantou | 120E51' 23N42' | IX | a | a | a | 9.24 |
| 406 | PI433406 | Shinyi, Nantou | 120E51' 23N42' | IX | a | a | a | 9.15 |
| 407 | PI433407 | Shinyi, Nantou | 120E51' 23N42' | IX | a | a | a | 8.40 |
| 408 | PI433408 | Shinyi, Nantou | 120E51' 23N42' | IX | a | a | a | 7.80 |
| 409 | PI433409 | Shinyi, Nantou | 120E51' 23N42' | IX | a | a | a | 5.58 |
| 410 | PI433410 | Shinyi, Nantou | 120E51' 23N42' | IX | a | a | a | 8.17 |
| 411 | PI433411 | Shinyi, Nantou | 120E51' 23N42' | IX | a | a | a | 5.53 |
| 412 | PI433412 | Shinyi, Nantou | 120E51' 23N42' | IX | a | a | a | 6.38 |
| 413 | PI433413 | Shinyi, Nantou | 120E51' 23N42' | IV | a | a | a | 1.17 |
| 415 | PI433415 | Shinyi, Nantou | 120E51' 23N42' | IV | a | a | a | 2.45 |
| 416 | PI433416 | Shinyi, Nantou | 120E51' 23N42' | IV | a | a | a | 1.85 |
| 417 | PI433417 | Shinyi, Nantou | 120E51' 23N42' | I | a | a | a | 15.82 |
| 418 | PI433418 | Shinyi, Nantou | 120E51' 23N42' | IX | a | a | a | 9.11 |
| 419 | PI433419 | Shinyi, Nantou | 120E51' 23N42' | I | a | a | a | 15.26 |
| 420 | PI433420 | Shinyi, Nantou | 120E51' 23N42' | IX | a | a | a | 11.06 |
| 421 | PI433421 | Shinyi, Nantou | 120E51' 23N42' | IX | a | a | a | 8.48 |
| 422 | PI433422 | Shinyi, Nantou | 120E51' 23N42' | IV | a | a | a | 1.47 |
| 424 | PI433424 | Shinyi, Nantou | 120E51' 23N42' | IX | a | a | a | 8.36 |
| 425 | PI433425 | Shinyi, Nantou | 120E51' 23N42' | IV | a | a | a | 1.80 |
| 426 | PI433426 | Shinyi, Nantou | 120E51' 23N42' | IX | a | a | a | 8.44 |
| 427 | PI433427 | Shinyi, Nantou | 120E51' 23N42' | IX | a | a | a | 9.64 |
| 428 | PI433428 | Shinyi, Nantou | 120E51' 23N42' | IV | a | a | a | 1.05 |
| 429 | PI433429 | Taimali, Taitung | 121E00' 22N37' | IV | a | a | a | 1.10 |
| 430 | PI433430 | Taimali, Taitung | 121E00' 22N37' | I | a | a | a | 10.25 |
| 431 | PI433431 | Taimali, Taitung | 121E00' 22N37' | I | a | a | a | 10.31 |
| 432 | PI433432 | Taimali, Taitung | 121E00' 22N37' | I | a | a | a | 9.05 |
| 433 | PI433433 | Taimali, Taitung | 121E00' 22N37' | IV | a | a | a | 0.80 |
| 434 | PI433434 | Taimali, Taitung | 121E00' 22N37' | IV | a | a | a | 1.51 |
| 435 | PI433435 | Taimali, Taitung | 121E00' 22N37' | IV | a | a | a | 1.76 |
| 436 | PI433436 | Taimali, Taitung | 121E00' 22N37' | IV | a | a | a | 1.35 |
| 437 | PI433437 | Taimali, Taitung | 121E00' 22N37' | IV | a | a | a | 1.65 |
| 438 | PI433438 | Daren, Taitung | 121E53' 22N27' | IV | a | a | a | 1.74 |
| 439 | PI433439 | Daren, Taitung | 121E53' 22N27' | IV | a | a | a | 1.23 |
| 440 | PI433440 | Daren, Taitung | 121E53' 22N27' | IV | a | a | a | 0.69 |
| 441 | PI433441 | Daren, Taitung | 121E53' 22N27' | IV | a | a | a | 1.78 |
| 443 | PI433443 | Lanyu, Taitung | 121E33' 22N25' | III | a | a | a | N. A. |
| 444 | PI433444 | Lanyu, Taitung | 121E33' 22N25' | III | a | a | a | 9.01 |
| 445 | PI433445 | Lanyu, Taitung | 121E33' 22N25' | III | a | a | a | 7.78 |
| 446 | PI433446 | Lanyu, Taitung | 121E33' 22N25' | III | a | a | a | 10.25 |
| 447 | PI433447 | Lanyu, Taitung | 121E33' 22N25' | III | a | a | a | 9.62 |
| 448 | PI433448 | Haiduan, Taitung | 121E10' 23N06' | IX | a | a | a | 7.94 |
| 449 | PI433449 | Haiduan, Taitung | 121E10' 23N06' | IX | a | a | a | 2.32 |
| 450 | PI433450 | Haiduan, Taitung | 121E10' 23N06' | IV | a | a | a | 1.49 |
| 451 | PI433451 | Haiduan, Taitung | 121E10' 23N06' | IX | a | a | a | 6.96 |
| 455 | PI433455 | Haiduan, Taitung | 121E10' 23N06' | IX | a | a | a | 10.22 |
| 456 | PI433456 | Haiduan, Taitung | 121E10' 23N06' | IX | a | a | a | 7.45 |
| 457 | PI433457 | Beinan, Taitung | 121E07' 22N47' | IV | a | a | a | 2.24 |
| 458 | PI433458 | Beinan, Taitung | 121E07' 22N47' | IV | a | a | a | 1.94 |
| 459 | PI433459 | Beinan, Taitung | 121E07' 22N47' | IV | a | a | a | 1.36 |
| 460 | PI433460 | Beinan, Taitung | 121E07' 22N47' | IV | a | a | a | 2.58 |
| 461 | PI433461 | Wutai, Pingtung | 120E43' 22N45' | IV | a | a | a | 1.70 |
| 462 | PI433462 | Wutai, Pingtung | 120E43' 22N45' | IV | a | a | a | 1.26 |
| 463 | PI433463 | Wutai, Pingtung | 120E43' 22N45' | I | a | a | a | 8.11 |
| 464 | PI433464 | Renai, Nantou | 121E07' 24N01' | IV | a | a | a | 2.03 |
| 465 | PI433465 | Renai, Nantou | 121E07' 24N01' | IX | a | a | a | 3.66 |
| 466 | PI433466 | Renai, Nantou | 121E07' 24N01' | IV | a | a | a | 2.60 |
| 467 | PI433467 | Renai, Nantou | 121E07' 24N01' | I | a | a | a | 16.92 |
| 468 | PI433468 | Renai, Nantou | 121E07' 24N01' | I | a | a | a | 16.82 |
| 469 | PI433469 | Haiduan, Taitung | 121E10' 23N06' | IX | a | a | a | 10.97 |
| 470 | PI433470 | Haiduan, Taitung | 121E10' 23N06' | IX | a | a | a | 10.06 |
| 471 | PI433471 | Haiduan, Taitung | 121E10' 23N06' | IX | a | a | a | 4.92 |
| 472 | PI433472 | Haiduan, Taitung | 121E10' 23N06' | IX | a | a | a | 5.80 |
| 473 | PI433473 | Haiduan, Taitung | 121E10' 23N06' | IX | a | a | a | 10.77 |
| 475 | PI433475 | Haiduan, Taitung | 121E10' 23N06' | IV | a | a | a | 1.26 |
| 476 | PI433476 | Haiduan, Taitung | 121E10' 23N06' | IV | a | a | a | 1.22 |
| 477 | PI433477 | Haiduan, Taitung | 121E10' 23N06' | IX | a | a | a | 8.65 |
| 478 | PI433478 | Haiduan, Taitung | 121E10' 23N06' | IX | a | a | a | 5.97 |
| 479 | PI433479 | Haiduan, Taitung | 121E10' 23N06' | IV | a | a | a | 3.29 |
| 480 | PI433480 | Haiduan, Taitung | 121E10' 23N06' | IX | a | a | a | 3.22 |
| 481 | PI433481 | Haiduan, Taitung | 121E10' 23N06' | IX | a | a | a | 8.66 |
| 488 |  | N. A. | N. A. | IX | a | a | a | 11.25 |
| A261 |  | SPNP, Taichung | 121E03' 24N28' | I | a | a | a | 8.24 |
| A280 |  | N. A. | N. A. | I | a | a | a | 10.95 |
| DC-1 |  | Wutai, Pingtung | 120E43' 22N45' | I | a | a | a | N. A. |
| DL-1 |  | Laiyi, Pingtung | 120E39' 22N31' | IV | a | a | a | 2.16 |
| DL-2 |  | Laiyi, Pingtung | 120E39' 22N31' | IV | a | a | a | 1.16 |
| DN-1 |  | Maolin, Kaohsiung | 120E40’ 22N54’ | I | a | a | a | N. A. |
| DNA-1 |  | Beinan, Taitung | 121E07' 22N47' | IV | a | a | a | 1.39 |
| DNI-1 |  | Dawu, Taitung | 121E54' 22N21' | I | a | a | a | 5.48 |
| HY-1 |  | Sandimen, Pingtung | 120E39' 22N43' | I | a | a | a | 14.52 |
| HY-2 |  | Sandimen, Pingtung | 120E39' 22N43' | IV | a | a | a | 1.30 |
| HY-3 |  | Sandimen, Pingtung | 120E39' 22N43' | IV | a | a | a | 2.05 |
| HY-4 |  | Sandimen, Pingtung | 120E39' 22N43' | IV | a | a | a | 1.86 |
| Ian-1 |  | Datong, Yilan | 121E31' 24N36' | IV | a | a | a | N. A. |
| Ian-2 |  | Nanau, Yilan | 121E48' 24N28' | IV | a | a | a | N. A. |
| LC-1 |  | Beinan, Taitung | 121E07' 22N47' | IV | a | a | a | 1.39 |
| LC-2 |  | Beinan, Taitung | 121E07' 22N47' | IX | a | a | a | 7.46 |
| LC-3 |  | Beinan, Taitung | 121E07' 22N47' | IV | a | a | a | 1.87 |
| LC-4 |  | Beinan, Taitung | 121E07' 22N47' | IV | a | a | a | 1.47 |
| LC-5 |  | Beinan, Taitung | 121E07' 22N47' | IV | a | a | a | 2.04 |
| ML-1 |  | Maolin, Kaohsiung | 120E40' 22N54' | I | a | a | a | 15.20 |
| ML-2 |  | Maolin, Kaohsiung | 120E40' 22N54' | I | a | a | a | 14.11 |
| ML-3 |  | Maolin, Kaohsiung | 120E40' 22N54' | IV | a | a | a | N. A. |
| NMS-1 |  | Namasia, Kaohsiung | 120E41' 23N13' | IV | a | a | a | N. A. |
| SMCS-1 |  | Jianshih, Hsinchu | 121E12' 24N43' | IV | a | a | a | 1.56 |
| SMCS-2 |  | Jianshih, Hsinchu | 121E12' 24N43' | IV | a | a | a | 1.78 |
| TTS-1 |  | TTDARES, Taitung | 121E09' 22N45' | IV | a | a | a | 1.83 |
| TTS-5 |  | TTDARES, Taitung | 121E09' 22N45' | IX | a | a | a | 2.98 |
| TU-1 |  | Taitung | 121E06' 22N73' | IV | a | a | a | 1.79 |
| WT-1 |  | Wutai, Pingtung | 120E43' 22N45' | IV | a | a | a | 1.15 |
| WT-3 |  | Wutai, Pingtung | 120E43' 22N45' | IV | a | a | a | N. A. |
| WT-4 |  | Wutai, Pingtung | 120E43' 22N45' | IV | a | a | a | N. A. |

^a^TTDARES: Taitung District Agricultural Research and Extension Station, COA; TDAIS: Taichung District Agricultural Research and Extension Station, COA; SPNP: Shei-Pa National Park; N. A. indicates the origin of the accession was not recorded.

^b^The coordinates are based on the location of the townships because the foxtail millet townships were dated where the seeds were collected from or requested from the locals. N. A. indicates the origin of the accession was not recorded.

^c^’a’ indicates PCR product was amplified with the expected size; I, III, IV, and IX represent *Wx* genotypes that determined by the amplified fragment of ex1/ex2 region.

^d^Apparent amylose content of corresponding accessions. na, accessions had insufficient mature seeds for AAC assay.
