# Supplementary material for: Waxy allele diversification in foxtail millet (Setaria italica) landraces of Taiwan
Source: PLoS One. 2018 Dec 31;13(12):e0210025. doi: 10.1371/journal.pone.0210025 (PMC6312202; doi:10.1371/journal.pone.0210025)
Supplement: S2 Table — (DOCX) [file pone.0210025.s004.docx]

**S2 Table. Primers used to assess *Wx* genotypes.**

| Primer name | Sequence (5’ to 3’) | Reference |
| --- | --- | --- |
| ex1 | F’ TgCAAgCCAgTgACggATCgACgACgACAC | Kawase *et al*. 2005 |
| ex2 | R’ ATgCCggTgACCAgCgTggAgggCTAgCTA | Kawase *et a*l. 2005 |
| ex2int2 | F’ CATggCCgTAAgTCCCATCgATCgATCATC | Kawase *et al*. 2005 |
| ex4r | R’ TAgCAgTggAAgAACCTCACCCTCTCGTAC | Kawase *et al.* 2005 |
| ex7 | F’ AgCAACTACCAgTCCAACgg | This study |
| ex10 | R’ TgTATAgCCACAgCACTCgg | This study |
| M7 | F’ CACCAgCCgCTTCgAgCCCT | Umeda *et al.* 1991 |
| R9 | R’ gCgAgCggCgCgATCTCCTC | Fukunaga *et al*. 2002 |
