# Supplementary material for: Waxy allele diversification in foxtail millet (Setaria italica) landraces of Taiwan
Source: PLoS One. 2018 Dec 31;13(12):e0210025. doi: 10.1371/journal.pone.0210025 (PMC6312202; doi:10.1371/journal.pone.0210025)
Supplement: S3 Table — (DOCX) [file pone.0210025.s005.docx]

**S3 Table. List of foxtail millet accessions studied by accession, *Wx* allele, TE insertion, and apparent amylose content.**

| Allele ^b^ | TE ^b^ | Length of TE ^*^ (bp) | Number of accessions | Accession ^a^ | Phenotype ^b^ | Range of AAC (%) | Avg. AAC ^c^ (%) |
| --- | --- | --- | --- | --- | --- | --- | --- |
| I |  |  | 21 | 385, 387, 397, 398, 400, 417, 419, 430, 431, 432, 463, 467, 468, DNI-1, ML-1, ML-2, A261, A280, HY-1, DN-1 ^**^, DC-1 ^**^ | non-waxy | 5.42-16.92 | 11.91±3.81 ^a^ |
|  |  |  |  |  |  |  |  |
| III | TSI-6/TSI-3 | 4,050 | 5 | 443^**^, 444, 445 ,446 ,447 | low AC | 7.78-10.25 | 9.16±1.05 ^ab^ |
|  |  |  |  |  |  |  |  |
| IV | TSI-2 | 5,250 | 61 | 383, 384, 386, 388,389, 390, 392, 393, 394, 395, 396, 413, 415, 416, 422, 425, 428, 429, 433, 434, 435, 436, 437, 438, 439, 440, 441, 450, 457, 458,459, 460, 461, 462, 464, 466, 475,476, 479, DL-1, DL-2, DNA-1, HY-2,HY-3, HY-4,TTS-1, SMCS-1, SMCS-2, WT-1, LC-1, LC-3, LC-4, LC-5, TU-1, 382^**^, Ian-1^**,^ Ian-2^**^, ML-3 ^**^,WT-3^**^, WT-4 ^**^, NMS-1 ^**^ | waxy | 0.69~3.29 | 1.61±0.5 ^c^ |
|  |  |  |  |  |  |  |  |
| IX | TSI-3 | 2,823 | 37 | 391, 399, 401, 402, 403, 405, 406, 407, 408, 409, 410, 411, 412, 418, 420, 421, 424, 426, 427, 448, 449, 451, 455, 456, 465, 469, 470, 471, 472, 473, 477,478, 480, 481,488, LC-2, TTS-5 | low AC | 2.32-11.25 | 7.6±2.42 ^b^ |

^a^ Accessions having insufficient mature seeds for AAC assay are indicated by **.

^b^ The classification is based on Kawase *et al*. (2005)

^c^ Means with different letter superscript are significantly different at 5% level, according to Fisher LSD test.
